# Typhoid Fever

**Published:** 1884-10

**Authors:** F. M. Brantley

**Affiliations:** Senoia, Ga.


### TYPHOID FEVER.*

BY F. M. BRANTLEY, M.D,, SENOIA, GA.

Idiopathic fevers, as taught by medical authors, embrace such a diversified nomenclature that the medical student can hardly be expected to retain in memory anything approximating to a clear view of the true nature and etiology of this class of diseases: For instance, authors tell us of and enumerate intermittent, remittent, continued tertian, double tertian, quotidian, congestive, yellow, bilious, ague, dengue, hemorrhagic, *et id omne genus*, and again typhus, typhoid, typho-malarial, typhoid pneumonia, besides many others said to be mongrel, sired by one cause and brought forth by another. Now these fevers have but two causes that produce them, and yet owing to adventitious circumstances make the grand array diversified as seems *ad infinitum*. all cooked up to suit the taste of the most fastidious, and before you have perused half that is said by way of division and subdivision you doubtless are ready as I was to conclude that their name is legion.

But when we come to sum up the whole matter and take a correct view in the premises, there are to be found only two causes which produce idiopathic fevers, and hence only two kinds; all others are only modifications of these, induced by adventitious circumstances.

One class is produced by malaria from vegetable changes or what is called decomposition of vegetable matter, and the other is in like manner produced by animal effluvia. The former has its antidote, the latter has none, so far as science has been able to reveal.

This then is the subject which we propose to discuss. Both of these fevers have some similarity, yet they are radically dissimilar in cause and effect; the one invariably has its ups and downs, while the other pursues and perseveres in the even tenor of its ways. These diversities are the great turning point upon which hinges the most important feature to the medical student, which is to arrive at a correct diagnosis, and here, perhaps, rests the great problem in success—early diagnosis has saved its thousands, while later diagnosis has lost its thousands.

Typhoid or typhus fever, both the offshoot of the same cause, have no known antidote, and when once contracted and diffused through the tissues must there abide, producing its peculiar routine

*Read before the Georgia Western Medical Association of symptoms in all its varied modifications, until it is eliminated from the system through the various outlets; a tedious process, and one rarely hurried or benefited by extraneous influences. Here let me say that I pity the man who is vain enough to assert his ability to cure typhoid fever. He may pilot his patient through the long perils of danger and perhaps be the means of averting death by his vigils and sagacity, yet he is doomed to the humiliating acknowledgment that he knows no curative remedy.

The causes of typhoid fever, as I before have said, are to be found wherever there are excreta from animal emanations, especially human; or cesspools often thrown from wastage from the culinary departments, to be found neglected about almost every household. These and the crowding together especially at night in illy ventilated apartments are the principal sources of this terror to mankind. We believe there is no disease to which humanity is heir that can show such fearful mortality; hence we should be on the alert to do all that enlightened science and experience can accomplish to avert its ravages. As I before hinted, an early diagnosis is the *sine qua non* in the successful treatment, and to that point I now call attention, because it is of vast importance to know and thereby hold in reserve the vital forces so as to be able to meet the final struggle with disease and wasted and exhausted vitality. The only and principal disease that we are likely to get mixed on is the miasmatic fevers first enumerated, which have their antidote and are but little feared; but they have their paroxysms both in animal heat and pulsations, which are so plain and palpable that the most obtuse observer cannot fail to detect them. But when we come to suspect typhoid fever, the constant pulse and animal heat are harbingers and soon reveal to the acute observer the insidious messenger in no ordinary meaning. The pulse maintains a constant uniformity in number of beats beyond all other fevers, and rarely varies in numbers beyond five beats in a day, and the same uniformity appertains in animal heat. These symptoms and others usually enumerated are enough to open the eyes of the practitioner and induce him to hold up on administration of medicines, and turn nurse, because good nursing now becomes the main question at issue. I do not mean by this that the doctor can be dispensed with, but he must possess, in an eminent degree, the faculty of both doctor and nurse. From what has been said the treatment may be somewhat foreshadowed, no definite plan as to minutiæ can be laid down, because complications will come, as is said in holy writ, "offenses must needs come," and in this disease they often come by means of good offers unintended but nevertheless often disastrous to our hopes and well grounded apprehensions. Therefore the guards should stand well to their duty, never allowing the least variation from suggestions from outsiders.

Unlike many other ills this is specially obnoxious to cathartics. Laxatives are often admissible and I dare say beneficial in the early stages of the fever, but are wholly interdicted later, but emollient enemata may be tolerated almost through the whole course. I have often seen patients who were convalescing turned back several days by one moderate evacuation induced by the mildest aperient, the pulse rising from 80 to 120 and remaining up for many days. This phenomena is not to be wondered at when we regard the pathology of this disease; the enteric lesions are too dangerous to be tampered with and can ill afford to be disturbed. Much suffering may be prevented and many dangers be avoided by a judicious and cautious treatment and regimen. A large proportion of cases need such diet as is nourishing and free from irritation, taken in small quantities and at stated intervals, and when the stomach is empty gum arabic will suffice to allay gastric irritation and keep the tongue soft and moist. To procure rest especially at night, four or five grains of camphorated powder will insure rest and ease; the formula I use is 300 grains each of camphor, creta preparata, pulverized licorice root, and 17 grains of sulphate morphia thoroughly mixed. To reduce the excess of animal heat nothing beats cold water applied externally with a sponge, this will control the heat most effectually. Another desideratum is to reduce the action of the heart and hence the number of pulsations. Norwood's veratrum has long been used for this purpose with varied effects and often unsuccessfully, its irritating properties often produce nausea to such a degree as to prevent its use altogether, and many medical men have almost dispensed with its use, especially in this disease.

Now after long years of observation I come to venture a substitute for veratrum, which has called forth this communication—that *brandy* is *the remedy*. I have to say that when properly administered it is the safest, surest and most effectual remedy; in doses from 6 to 30 drops given regularly every two hours, it will almost invariably reduce the pulse in a few hours from 120 to 80. Its effects are so marvelous that I was long in accepting the results from the use of the spirits. It appears to supply the system with the necessary carbon and in a great measure relieves the tissues and supplies material for the disease to prey upon. Although prejudiced in the extreme to alcohol, I am forced to make terms with it in this ailment, and think that it is rarely necessary to give over ten drop doses; its use indiscriminately doubtless has often proved worse than useless in the treatment of typhoid fever. The tympanites so often complained of and for which turpentine and many other nauseating remedies, together with blisters, have so often been used, is ordinarily nothing but wind or gas in the large bowels and can be expelled and prevented by carminatives. This concludes all I have to say on this subject at this time.

